# Compound Heterozygous Variants in the Coiled-Coil Domain Containing 40 Gene in a Chinese Family with Primary Ciliary Dyskinesia Cause Extreme Phenotypic Diversity in Cilia Ultrastructure

**DOI:** 10.3389/fgene.2018.00023

**Published:** 2018-02-02

**Authors:** Lin Yang, Santasree Banerjee, Jie Cao, Xiaohong Bai, Zhijun Peng, Haixia Chen, Hui Huang, Peng Han, Shunyu Feng, Na Yi, Xueru Song, Jing Wu

**Affiliations:** ^1^Centre for Reproductive Medicine, Tianjin Medical University General Hospital, Tianjin, China; ^2^BGI Genomics, BGI-Shenzhen, Shenzhen, China; ^3^Department of Respiratory, Tianjin Medical University General Hospital, Tianjin, China; ^4^Department of Radiology, Tianjin Medical University General Hospital, Tianjin, China

**Keywords:** *CCDC40*, PCD, targeted next-generation sequencing, mRNA expression, intracytoplasmic sperm injection

## Abstract

**Purpose:** Primary ciliary dyskinesia (PCD) is a rare genetic disorder manifested with recurrent infections of respiratory tract and infertility. Mutations in more than 20 genes including the Coiled-Coil Domain Containing 40 (*CCDC40*) gene are associated with PCD. A Chinese proband with a clinical diagnosis of PCD was analyzed for mutations in these genes to identify the genetic basis of the disease in the family. The proband showed altered mucociliary clearance of the airways, various degree of hyperemia and edema of the mucous membrane, left/right body asymmetry, infertility and ultrastructural abnormality of cilia in both sperm and bronchioles.

**Methods:** The DNA from the proband was analyzed for genetic variation in a subset of genes known to cause PCD using targeted next generation sequencing in order to understand the molecular and genetic basis of the PCD in present family. The result of targeted next generation sequencing has been validated by Sanger sequencing and q-PCR.

**Results:** Targeted next-generation sequencing identified two novel mutations (c.1259delA and EX17_20 deletion) in *CCDC40* gene that causes abnormal *CCDC40* mRNA expression. These two novel variants cause disorganization of axoneme filaments, which resulted in reduction of sperm motility and phenotypic diversity in ultrastructure of cilia in the proband.

**Conclusion:** These findings highlight the significance of the mutations in *CCDC40* as novel candidates for genetic testing in PCD patients as well as the key role of ICSI treatment for the families affected by this ciliary dysmotility. Our findings showed that our work enriched the performance of cilia ultrastructure which were not previously reported in PCD patients.

## Introduction

Primary ciliary dyskinesia (PCD) [MIM# 613808] is a rare disorder characterized by abnormal structure and function of motile cilia, including translocation, reduction or complete loss of the dynein arms or axoneme structure, with autosomal recessive mode of inheritance and X-linked form inheritance. (MIM# 300991). PCD has a higher incidence (∼20–30%) in people related by blood and relatively lower incidence (∼7–9%) in compatriots (Meeks and Bush, 2000). Clinical diagnosis is determined by the presence of classical PCD phenotypes; i.e., recurrent respiratory tract infections, bronchitis, rhinosinusitis, bronchiectasis, and infertility (Noone et al., 2004). The combination of bronchitis, chronic sinusitis and situs inversus is termed as Kartagener’s syndrome. PCD affects approximately 1:15,000–30,000 live births (Bush et al., 2007), with extreme phenotypic heterogeneity. The symptoms are often present since the birth (Barbato et al., 2009) and the incidence is non-specific between genders.

Mutations in genes associated with abnormal axonemal assembly and function are responsible for the clinical manifestation of PCD cases (Linck et al., 2016). To date, 33 genes have been identified to be associated with autosomal recessive PCD and one PIH1D3 gene caused X-Linked PCD (MIM# 300991) (**Supplementary Data Sheet S1**). The genes most commonly identified to result in PCD were DNAH5 (15–21%), DNAI1 (2–9%), both of which code for the structural components of the outer dynein arms; DNAAF1 (LRRC50) (4–5%), which code for the structural components of the outer and inner dynein arms; CCDC39 (2–10%), DNAH11 (6%), and LRRC6 (3%) (Kano et al., 2016). However, strong genotype-phenotype correlation has been shown among PCD patients. Genetic analysis reported that about 12% of PCD cases showed perturbed 9+2 microtubule cilia structure and subsequent loss of inner dynein arm (IDA) (Antony et al., 2013). In addition, approximately, 4–8% of all PCD cases result from mutations in *CCDC40* gene (Kano et al., 2016). *CCDC40* is expressed in tissues that contain cilia, which physically interacts with the other axonemal components and serve as a part of the axoneme structural scaffold which is essential for normal movement of cilia. Mutations of *CCDC40* causes ultrastructural defects in cilia characterized by the misplacement of the microtubular doublets structure along with irregular assembly of dynein arms (DAs) and lack of radial spokes complexes, leading to axonemal disorganization (Becker-Heck et al., 2011). *CCDC40*-mutant cilia often showed loss of waveform and become completely immotile.

Complete loss of sperm motility in patients with PCD results in infertility, and pregnancy under normal conditions becomes impossible (Zariwala et al., 1993). Therefore, intracytoplasmic sperm injection (ICSI) is the best possible tool for male patients with PCD to obtain healthy offspring. The hypo-osmotic swelling test (HOST) is used to select immotile spermatozoa prior to ICSI in several cases as well.

In this study, we systematically performed diagnostic tests and applied a combination of NGS and Sanger sequencing to identify the genetic cause of PCD in this Chinese family. We also found phenotypic diversity in ultrastructure of cilia, not only in the respiratory tract between the patients carrying the same mutations, but also among different parts of respiratory tract and sperm in the proband. This diversity was not previously reported in PCD patients.

## Materials and Methods

### Patients and Clinical Materials

Written informed consent was obtained from all family members participating in this study. The project was approved by the ethics committee of the Tianjin Medical University General Hospital and was performed in accordance with the Principles of the Declaration of Helsinki.

### Radiological Examination

In order to understand the effect of these two novel mutation on respiratory system, radiological examinations were taken in the proband and proband’s sister. High resolution computed tomography (HRCT) has been performed using 128 row multi-slice Computed tomography (CT) (SIMENS SOMATOM Definition Flash CT, German), slice 1 mm, space 10mm, FOV256. Magnetic resonance imaging (MRI) of paranasal sinus was performed using 1.5T machine (GE MRI 360, United States) using four channels head and neck unite coil.

### Bronchoscopy Examination

Bronchoscopy examination was performed for the diagnostic and therapeutic purposes.

### Bronchial Cilia and Sperm Flagellum Electron Microscopy Examination

In order to understand the abnormality in the ultrastructure of bronchial cilia, we used transmission electron microscope (TEM) examination in the proximal, distal and middle section of bronchial cilia. TEM analysis identified the ultrastructural abnormality of cilia (disarrangement, reduction, absence of DA and microtubular doublets structure). Conventional electron microscopy was performed as described previously (Wang et al., 2014).

### Targeted Next Generation Sequencing

DNA sample obtained from the proband (II-5) was subjected to carry out targeted gene-based next-generation sequencing. Roche NimbleGen’s (Madison, United States) custom Sequence Capture Human Array was designed to capture 76087 kb of targeted sequence, covering all the exons and flanking sequence (including the 100 bp of introns) of 20 genes which were known to be associated with PCD at the design phase of the target NGS panel (**Supplementary Data Sheet S1**). The average sequencing depth of the target area was 306.06 with 98.49% coverage. The procedure for preparation of libraries was consistent with standard operating protocols. All the detected variations in the 20 known genes can be seen in **Supplementary Data Sheet S2**.

### Sanger Sequencing and qPCR

To validate putative mutations in the family members I-1, I-2, II-1, and II-5, conventional Sanger sequencing (the primers: F5′- ACCACCTGGCACTACTTCAG -3′, R 5′- ATACAAGTTGACGCCACCCA -3′) and qPCR (the primers: *CCDC40* cds17 F5′- AACCTTTCAGAGATCGTGGC -3′, R 5′- GAGCGGAACAGGAACACGTA -3′; *CCDC40* cds20 F5′- CAACTGGCAAAAGAGATGCGT -3′, R 5′- CCTGTGGATCTCGCCCTTC-3′) were carried out. Primers were designed based on the Human Genome Sequence (GRCh38 primary assembly) and the reference sequence NM_017950 of *CCDC40* gene.

### The Analysis of *CCDC40* mRNA Relative Expression

Total RNA was extracted from the peripheral blood of the family members (I-1, I-2, II-1, II-5) as well as from healthy control by the use of the Trizol reagent according to manufacturer’s instructions. Subsequently, 1 μg of RNA was reverse-transcribed into cDNA using a first-strand cDNA synthesis kit (Takara, China). 1 μl of cDNA was used for quantification on an ABI 7500 real-time machine (ABI, CA, United States) using SYBR Green (Takara, China). GAPDH primers was used as the relative quantification of the internal reference gene and used for normalization of *C*t values.

### Ovarian Stimulation and ICSI Procedures

Oocyte collection was performed 36 h after triggering ovulation by Human Chorionic Gonadotrophin (HCG). Twenty-one of the oocytes were at the M-II stage and used for insemination through ICSI. On the day of oocyte retrieval, the sperm, obtained via masturbation, were processed using two-layer discontinuous density gradients, as no morphological criteria exist for vitality assessment, the HOST was proposed to perform ICSI. Other methods, such as FeNO test, hormone levels, sperm analysis, can be seen in **Supplementary Data Sheet S3**.

## Results

### Patients and Family

**FIGURE 1:**
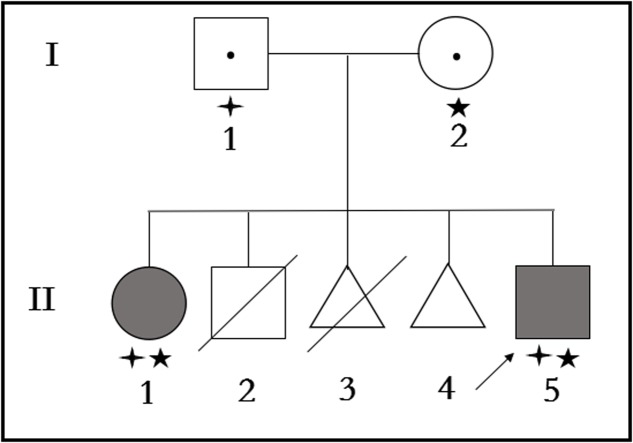
Pedigree structure of the family with Primary ciliary dyskinesia. Family members with Primary ciliary dyskinesia are indicated with shading. Squares and circles denoted males and females, respectively. Triangles denoted fetuses when II-3 was artificially aborted fetus, and II-4 was spontaneous aborted fetus. Individuals labeled with a slash were deceased. Individuals undetected or without gene mutation are indicated with no symbols. Roman numerals indicate generations. Arrow indicates the proband (II-5). Family members detected with *CCDC40* gene mutation (c.1259delA) are indicated with stars (aaa). Family members detected with *CCDC40* gene deletion (EX17_20del) are indicated by “four angled star” ([scale=.5]img001).

The proband is a 23 years old Chinese man having non-consanguineous parents (**Figure 1**). He is a non-smoker. The proband (II-5) has been referred to the Centre for Reproductive Medicine, Tianjin Medical University General Hospital, following 1 year of suffering from primary infertility. Family history showed the parents were non-consanguineous. No history of PCD, significant respiratory disease, infertility, or hearing impairment was identified in these family members except the proband’s sister (II-1).

### Clinical Features

The specific clinical manifestations of each patient in this family are shown in **Table 1**.

**Table 1:** Summary of the clinical features of the PCD family (II-1 ∼ II-5).

Characteristics	II-1	II-2	II-3	II-4	II-5
Gender	Female	Male	Unknown	Unknown	Male
Age	28 years	2 years	–	–	23 years
Birth status	Full-term natural labor with a weight of 2.6 kg	Full-term natural labor	Induced abortion	Spontaneous abortion	Full-term natural labor with a weight of 2.3 kg
Onset	Cough, blood in phlegm and gruff breathing, 1st month of birth	Cough, expectoration and fever, 1st month of birth	Unknown	Unknown	A moderate cough 3 days after birth
Respiratory symptom	Medium cough with expectoration of purulent	Severe respiratory symptoms	Unknown	Unknown	Medium cough with expectoration of purulent
Age of diagnosis of bronchial asthma and mirror-image reversal	1 year	–	–	–	21 years
Mentally competent	Yes	Cannot speak until his age of 2 years	Unknown	Unknown	Yes
Fertility problems	Primary infertility for 3y	Unknown	Unknown	Unknown	Primary infertility for 1 year
Smell problems	No	No	Unknown	Unknown	No
Hearing problems	No	No	Unknown	Unknown	No
History of dust, smoking or toxicant exposure	No	No	Unknown	Unknown	No
Respiratory function test	Obstructive ventilatory dysfunction with diffuse dysfunction	Unknown	Unknown	Unknown	Obstructive ventilatory dysfunction with diffuse dysfunction
FeNO	No abnormality	Unknown	Unknown	Unknown	No abnormality
FEV1/FVC	45%	Unknown	Unknown	Unknown	43%
FEV1	0.76	Unknown	Unknown	Unknown	0.92

*FeNO, fractional exhaled nitric oxide; FEV1, forced expiratory volume in 1 s; FVC, forced vital capacity*.

### Medical Examination

#### The Proband (II-5)

The proband (II-5) exhibited normal secondary sexual characteristics, with a height of 163 cm, weighing 67 kg. Cardiovascular examination found no abnormality. Both testicular volumes were 10 ml, firm feeling, the left epididymis was located in front of the testicles, the right epididymis and bilateral vas deferens were normal, and there was no evidence of varicocele.

**FIGURE 2:**
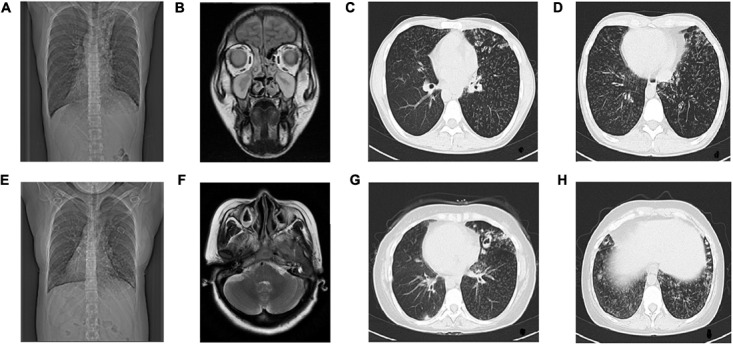
Radiological examination of Proband and Proband’s Sister. The radiological examination of Proband (II-5), **(A)** CT showed situs inversus. **(B)** Coronal view of paranasal sinus MRI shows bilateral maxillary sinusitis and ethmoid sinusitis. **(C,D)** HRCT of chest shows bronchitis and bronchiolitis. Bronchiectasia involves in left lobes. Centrilobular nodules and tree-in-bud sign distribute diffusely in both lobes.The radiological examination of Proband’s sister (II-1), **(E)** CT showed situs inversus, **(F)** Transverse view of sinus MRI showed bilateral maxillary sinusitis, **(G,H)** HRCT of chest showed bronchitis and bronchiolitis, ring-shaped or ductal opacities bronchiectasia involves in both lobes. Secretion obstruction can be seen in some saccular bronchiectasis in the upper left lobes.

Computed tomography of chest and MRI of sinus were performed. CT tomogram showed total situs inversus. Chest HRCT showed multiple cystic and double track bronchiectasis in the left lobe, quite similar clinical signs were seen in mucinous embolization. The other manifestations on HRCT were as follows: bronchial wall thickening, centrilobular nodules and tree-in-bud sign distributed diffusely in both lung lobes, which suggests bronchitis and bronchiolitis. Paranasal sinus MRI showed mucosal thickening in the bilateral maxillary sinus, ethmoid sinus, sphenoid sinus and frontal sinus, with iso-T1 and long-T2 signal (**Figures 2A–D**).

Hormone level test (FSH:7.92 IU/L, LH:7.53 IU/L, E:34.22 pg/ml, T:304 ng/dl) showed no abnormality. Karyotype (Verma and Babu, 1989) was 46, XY, Y chromosome AZF microdeletion detection covering common 6-site (SY84, 86, 127, 134, 254, 255), was not found abnormal.

Sperm analysis revealed a severe oligozoospermia, the concentrations was less than 1 × 10^6^/ml in several evaluations with complete immotile sperm. The sperm viability with Eosin-Y staining revealed a viability of 62%, while HOST was 52%. Morphologically, there was no normal sperm identified by Diff-quick staining. Most sperms were detected with tail deformities (short tails, lack of tails, curled tails), but the shape of the sperm head was good. Sperm germiculture showed no abnormality.

#### The Proband’s Sister (II-1)

She has been tested though chest CT and sinus MRI, same as the proband. The manifestations of her chest HRCT were total situs inversus, bronchiectasia, bronchial wall thickening, centrilobular nodules and tree-in-bud sign in both lung lobes, which is heavier than the proband in bronchiectasis. Her paranasal sinus MRI showed lighter pansinusitis than the proband. (**Figures 2E–H**).

### Bronchoscopy Examination

**FIGURE 3:**
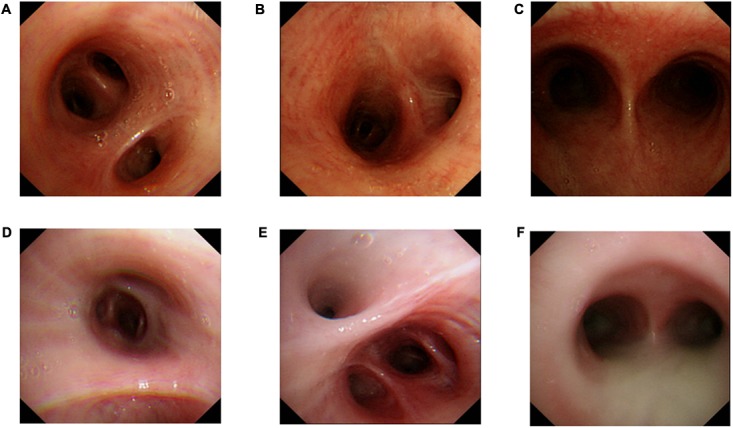
Bronchoscopy examination. Proband‘s (II-5) Bronchoscopy examination: **(A)** mucosal biopsy were performed on the proband’s right middle bronchus, **(B)** right lower lobe basal section of the bronchus, **(C)** carina of trachea. A small amount of purulent secretion is secreted by the tracheal carina. Bronchoscopy examination of the proband’s elder sister (II-1): **(D)** mucosal biopsy were performed on the elder sister’s right middle bronchus, **(E)** right lower lobe basal section of the bronchus, **(F)** carina of trachea. A large number of purulent secretion are secreted by the tracheal carina.

Bronchoscopy examination of the proband and his elder sister showed extensive hyperemia and edema of the mucous membrane (**Figures 3A–F**) from the main trachea to the segmental bronchi in both side with large amounts of purulent secretions (**Figure 3F**) and mirror inversion (**Figures 3A–F**). The congestive edema of mucous membrane of the proband’s elder sister is more apparent, with more purulent exudate (**Figures 3D–F**). Mucosa biopsies were performed at the main trachea carina, right middle bronchial trunk and right lower lobe basal bronchus, and 4 mucosa tissues were clamped at each site for pathological examination under electron microscope (**Figure 3**).

### Bronchial Ciliary Electron Microscopy Examination

#### Proximal Bronchial Ciliary Electron Microscopy of the Proband (II-5)

**FIGURE 4:**
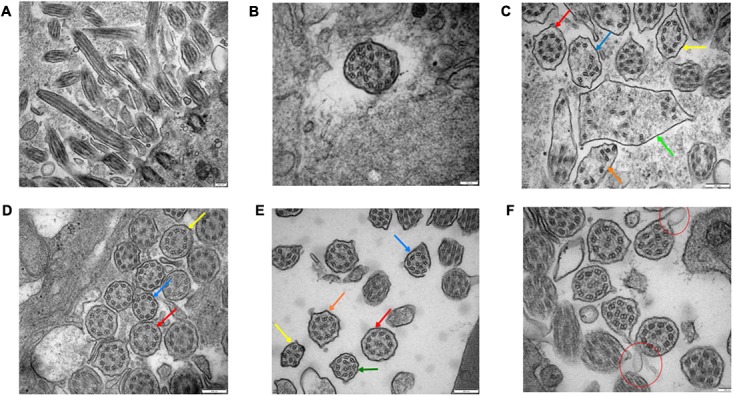
Bronchial ciliary electron microscopy of the proband (II-5). Proximal bronchial ciliary electron microscopy of the proband, **(A)** several microtubules in vertical section, microtubules run through from the top to the base, their relative location stay the same, **(B)** transverse section of the cilia, part of the cilia plasma membrane fusion to form composite cilia, **(C)** cilia in transverse section, red arrow:normal 9+2 structure, the radial spokes structures and DAs are faintly recognizable; yellow arrow: 9+0 structure, the position of peripheral microtubules are shifted, central microtubules disappear; blue and orange arrow:9+0 structure, the position of peripheral microtubules are completely irregular, central microtubules disappear, irregular shape of cross section; green arrow: composite cilia, irregular shape of cross section, one pair of central microtubules and several peripheral microtubules, complete confusion of position. Total chaos of radial spokes and DAs structures. **(D)** The distal bronchial mucosa cilia electron microscopy of the proband; red arrow: normal 9+2 structure, the radial spokes structures and DAs are recognizable; yellow arrow: the number and position of peripheral microtubules are normal, the central microtubules shifted; blue arrow: the position of one peripheral microtubule and central microtubule are shifted. **(E)** The middle section of bronchial mucosa cilia electron microscopy of the proband; red arrow: normal 9+2 structure, the radial spokes structures and DAs are faintly recognizable; yellow arrow: 8+0 structure, the number of peripheral microtubules are reduced, and position are completely irregular, central microtubules disappear; blue arrow: 8+2 structure, the number of peripheral microtubules are reduced, position of microtubules are shifted; orange arrow: 9+2 structure, the position of peripheral microtubules are shifted; green arrow: 9+2 structure, the position of all microtubules are shifted. **(F)** Bronchial ciliary electron microscopy of the proband’s sister showed more cells with oval synapses (Indicated by the red circle).

**FIGURE 5:**
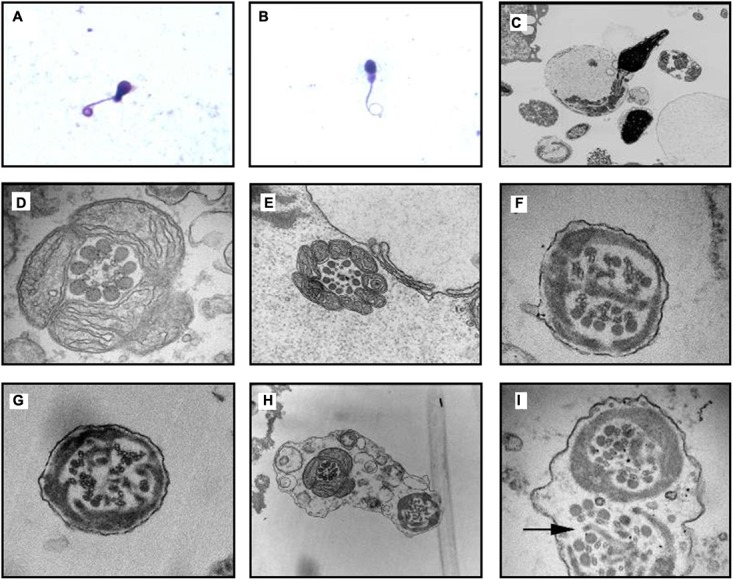
Transmission electron microscope (TEM) analyses of *CCDC40*-mutant sperm cilia ultrastructural abnormality from proband. **(A,B)** Optical microscope of Diff-quick stained sperm from the PCD patient indicated the curly tail of sperm. **(C)** Vertical section of a sperm, the heads are normal while the flagellum is flexural and encircled with the plasma membrane, notice the disarrangements of the mitochondria. **(D,E)** Cross section of mid piece region of flagellum with normal mitochondria packing at 98,000× magnification; morphology of outer dense fibers ODF(s) appeared normal, 1–3 ODFs are retained but mislocalised; absent central pairs with eccentric number and displacement of outer doublets; the radial spokes structures are reduced or absent; **(D)** also showed faintly visible derangements of IDA and ODA; **(E)** also showed absence of IDA and ODA. **(F,G)** Cross section of principal piece region of flagellum; abnormal quantities and disorganization of ODFs arrangement; central microtubule pair get lost, decreased in number with perturbed peripheral microtubular doublets structure and radial spokes, dynein arms are beyond recognition. Excess fibrous sheaths are observed. **(H,I)** Two section of the same flagellum move toward in the opposite direction, surrounded by the same outer membrane. **(H)** Showed cross section of mid piece (left) and principal piece (right), the perturbations of axoneme structure get more and more worse from the proximal to distal. **(I)** Showed that both the mid piece region of flagellum showed obvious structural abnormality, note the absence of a truncated lateral column (arrow), decreased central pair and acentric microtubular central pairs.

The basic structure of cilia in cytoplasm are various (**Figures 4A–C**), the microtubule arrangement (**Figure 4C**) is more disordered than the middle (**Figure 4E**) and distal segment (**Figure 4D**). Still, the overall structure is far better than the cilia of the sperm (**Figure 5**). It’s worth mentioning that compared to the cilia of the sperm, axoneme structure gradually disordered from the proximal (**Figures 4A–C**) to the distal part (**Figure 4D**) of the bronchioles, the microtubules of the bronchial cilia were less disordered (**Figures 4B–F**), the relative position of microtubules in the same cilium is fixed, not shifting from the proximal end to the distal end, indicating that the different transverse sections of the same cilia are basically the same (**Figures 4A–C**).

#### The Distal Bronchial Mucosa Cilia Electron Microscopy of the Proband (II-5) (**Figure 4D**)

The abnormity of the basic structure of cilia are less evident in cilia of distal bronchia, the polygonal cilia are less found, more cilia are close to circle. The arrangement of microtubules is more normal than that of the proximal bronchia (**Figures 4B,C**). The central microtubules were displaced but still can be identified in each cilium. The peripheral microtubules are basically nine pairs and the shift is not obvious. The overall structure is much better than the proximal bronchia (**Figures 4B,C**).

#### The Middle Section of Bronchial Mucosa Cilia Electron Microscopy of the Proband (II-5) (**Figure 4E**)

The middle section of bronchial mucosa cilia electron microscopy showed the abnormity of cilia is between the proximal (**Figures 4B,C**) and distal bronchia (**Figure 4D**).

#### The Bronchial Mucosa Cilia Electron Microscopy of the Proband’s Sister (II-1) (**Figure 4F**)

The proband’s sister’s (II-1) bronchial mucosa cilia change is slightly better than the proband’s (**Figures 4B–E**), other than the cells with oval synapses are more.

#### Transmission Electron Microscopic (TEM) Analysis of Sperm Cilia

Transmission electron microscopic analysis identified the ultrastructural abnormality of sperm cilia (disarrangement, reduction, absence of DA and microtubular doublets structure) (**Figure 5**).

#### Identification and Validation of Two Novel *CCDC40* Mutations

**FIGURE 6:**
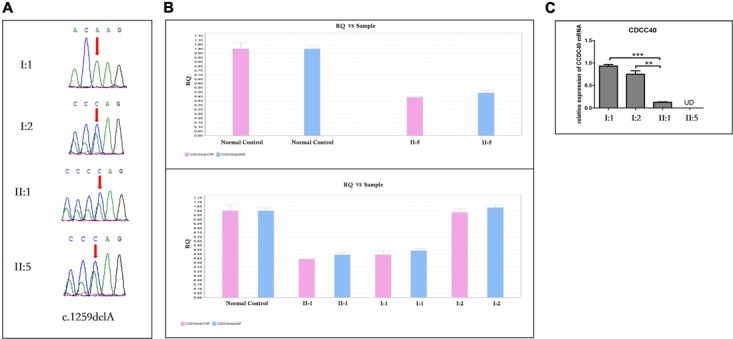
Sanger Sequencing, the qPCR results and the analysis of *CCDC40* mRNA relative expression (*n* = 3). **(A)** Sanger sequencing analysis of *CCDC40* mutation in the family. The deletion (c.1259delA) was observed in the mother (I:2), and inherited to both daughter (II:1) and son (II:5). The other deletion (EX 17_20del) was observed in the father (I:1), and inherited to both daughter (II:1) and son (II:5). **(B)** A large deletion (EX17_20del) was confirmed by qPCR in the family members (I:1, II:1 and II:5). **(C)** ^∗^ Stands for different statistical differences: ^∗^*P* < 0.05, ^∗∗^*P* < 0.01, ^∗∗∗^*P* < 0.001, and indicate the significant differences between groups. UD means “Undetermined,” when no data output can be detected from qPCR, it would reminder “undetermined.” However, here the “UD” should represent that mRNA expression was very low, which may relate to the RNA initial concentration of blood. “*N* = 3” means each blood sample was used to establish three qPCR amplification system, respectively. The *CCDC40* mRNA relative expression of both proband (II:5) and his sister (II:1), who carry two kinds of mutation, was apparently lower that the expression of their parents with normal phenotype.

Targeted NGS identified two novel mutations in the *CCDC40* gene: (i) a frameshift mutation (c.1259delA) in exon 8, inherited from his mother and resulted in a reading frameshift in a way that it translates to 421 valine followed by a premature stop codon at position 422 (p.Val421TrpfsX2), possibly leading to truncated protein; (ii) a EX17_20 deletion (**Supplementary Figure S1**), inherited from his father may result in damaging the function of coding the protein. c.1259delA was not reported in published literature and was neither found or very low frequency in NCBI dbSNP, 1000 human genome dataset, ExAC, genome AD and BGI’s database (**Supplementary Data Sheet S2**). The biological effects of c.1259delA was predicted by MutationTaster^1^ and HSF3.0^2^ as “Prediction disease causing” and “Potential alteration of splicing.” These two novel *CCDC40* mutations were confirmed by Sanger sequencing (**Figure 6A**) and qPCR (**Figure 6B**), respectively, in the family members (I-1, I-2, II-1, II-5).

#### The Analysis of *CCDC40* mRNA Relative Expression

The *CCDC40* mRNA relative expression of both proband (II:5) and his sister (II:1), who harbor both the mutations in *CCDC40* gene, was apparently lower than that of their carrier parents with normal phenotypes (**Figure 6C**).

## Discussion

In this study, we investigated a Chinese proband with PCD and identified two novel mutations in the *CCDC40* gene; a frameshift mutation (c.1259delA) and a EX17_20 deletion, inherited from his mother and father, respectively. These two novel *CCDC40* mutations were *loss-of-function* mutation, confirmed by Sanger sequencing and qPCR, respectively, in the family members.

In human, ultrastructural defects in cilia results in altered ciliary beat in PCD patients (Satir and Christensen, 2007; Matsumoto et al., 2010). PCD is a rare autosomal recessive genetic disease (Blouin et al., 2000), manifest with recurrent upper respiratory tract infections, increased morbidity in visceral, male infertility and female ectopic pregnancy or infertility (Boon et al., 2015). Since it is found to be associated in PCD, a total of 33 mutations in autosomal recessive *CCDC40* gene have been identified (Becker-Heck et al., 2011; Blanchon et al., 2012; Sui et al., 2016). To be more precise, 19% PCD patients have a complete absence of IDAs and 81% presented with a compound variation of absence of IDAs and axonemal disorganization (Papon et al., 2010). Researches showed that *CCDC40* protein plays a key role in assembling distinct coiled-coil domain-containing proteins complexes localized to the axoneme, including DNALI1-containing IDAs, GAS11-containing DRC and radial spokes, all of them eventually regulate the movement of a cilia and its left–right axis formation. In addition, *CCDC40* is necessary for proper interconnections among microtubules or serves as a docking domain. *CCDC40* mutated cells are lacking of some of the nexin-dynein regulatory complex structure (Becker-Heck et al., 2011; Blanchon et al., 2012; Antony et al., 2013).

In this study, we applied targeted gene-based NGS that has proven to be reliable, rapid and cost- effective, was capable of identifying the candidate mutation in patients with PCD (Djakow et al., 2016; Guo et al., 2017). According to American College of Medical Genetics and Genomics (ACMG) variant interpretation guidelines, these two mutations were identified as “likely pathogenic” mutations (Richards et al., 2015). Using protein modeling tools, the researchers found that *CCDC40* has 1142 residues and 8 predicted coiled-coils, *CCDC40* frameshift mutations are extremely common (Antony et al., 2013). The mutations we found in this study were located in two regions of putative protein functional domains and nine mutations previously published in two regions (shown in **Supplementary Figure S2**). We further analyzed the *CCDC40* mRNA relative expression between affected and unaffected members of this family. We found that the mRNA expression of patients carrying compound heterozygous mutations was significantly lower than that of healthy parents.

Lung function, radiological and bronchoscopy examination were performed (Frija-Masson et al., 2017) to understand the effect of these two novel mutation on respiratory system, we also used TEM to identify the ultrastructural abnormality of cilia. These findings provide compelling evidence that these two loss-of-function mutations are responsible for the proband and his sister.

There are more than 200 genes code for ciliary components, and any mutation of these genes may lead to the ultrastructural abnormalities of the cilia (Heuser et al., 2009). Patients with the same clinical/ultrastructural performance may not be harboring the same gene mutations, at the same time, mutations in distinct genes may result in the same clinical/ultrastructural abnormalities (Escudier et al., 2009; Bush and Hogg, 2012). This is why PCD is identified as a disease with high genotypic and phenotypic heterogeneity between patients. In our case, the clinical manifestations shared similar respiratory symptoms among the siblings, but differed from other PCD patients. Their respiratory illnesses were not typical or serious, the main clinical manifestation was coughing and purulent sputum production, no supplemental oxygen was required during their treatment. Unlike many pediatric patients with PCD were diagnosed with bronchial asthma at median age of 5.3 years (Bush and Hogg, 2012), the siblings were diagnosed when they came of age due to atypical clinical performance. It is very interesting to know that proband’s elder brother (II-2) died due to severe respiratory symptoms, accompanied by infectious fever. Though he was not tested for *CCDC40* gene, if the proband’s elder brother (II-2) was also a patient with the *CCDC40*-mutation, his clinical manifestations were very different from the proband (II-5) and his elder sister (II-1), which further shows the heterogeneity of *CCDC40* associated PCD.

In addition, no significant change in the length of cilia was identified in respiratory cilia from PCD patients carrying mutations in *CCDC40* gene, implying that mutant *CCDC40* protein disrupted ciliary movement by gross ultrastructural defects (Becker-Heck et al., 2011). It is evident from the cross-section, that abnormal changes in the morphology of the sperm flagella are the most serious (**Figure 5**), resulting in sperm that is 100% immotile. By contrast, cilia with normal structure emerge in specimens from three different level (the first, second, third bronchial levels) of the siblings. From the longitudinal section, the bronchial ciliary axoneme structure getting worse from the proximal to distal (**Figures 4B–E**), but the microtubules of the bronchial cilia are relatively fixed (**Figures 4A–F**), such structures, though abnormal, may have limited impact on ciliary coordination. Therefore, the abnormal performance of the respiratory system of the proband is much less than that of the reproductive system. Adults with PCD is heterogeneous and usually moderate in respiratory function, but appears more severe in women (Frija-Masson et al., 2017). The abnormality of lung function in the siblings is similar, but the sister is heavier than proband in bronchiectasis and lung infection. Compared to the proband (**Figures 3A–C**), the congestive edema of mucous membrane of the elder sister is more apparent (**Figures 3D–F**) with more purulent exudate and larger amount of purulent secretion are secreted in the tracheal carina (**Figure 3F**). To our surprise, the bronchial ciliary electron microscopy performance of the sister was slightly better than the proband; we speculate that this is because the ciliary membrane protrudes outward and extends into a tubular structure. This phenomenon has not been reported in the literature.

Viscera under normal circumstances should rotate to the right during the 10th to 15th week of pregnancy. Left-right patterning reversal of all internal organs with no apparent physiological consequences is randomized in about half of individuals with PCD due to the nodal cilia loss normal swing during embryonic development. *CCDC40* protein is specifically expressed in the embryonic node and required for formation or maintenance of cilia (Becker-Heck et al., 2011). Coincidentally, in our study, both the siblings showed defect in organ laterality which has not been reported before in Chinese population.

**FIGURE 7:**
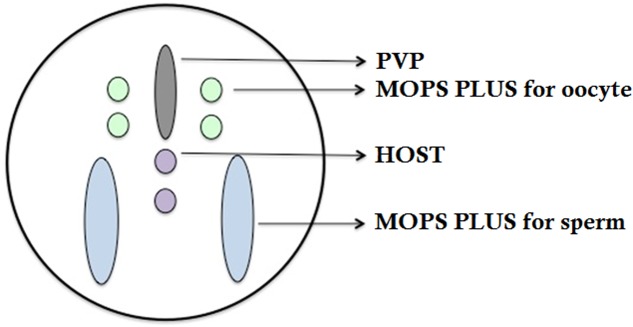
Working dish for ICSI procedure.

The clinical outcome of ICSI is significantly related with the vitality and morphology of sperm. Hence, the key to success in ICSI for patients with PCD is the selection of viable sperms (**Figure 7**). Relevant literature found in (Esteves et al., 2017), showed that injection of both epididymal/testicular-extracted and ejaculated sperm have been proven to be effective and does not adversely affect the development of the resulting embryo. HOST is a classic method recommended by WHO for selecting viable sperm. It is a simple, reliable and non-damaging technique that allows us to select of viable spermatozoon prior to oocyte injection. Here, the proband has presented with completely immotile sperm, which does not respond to any medical therapies.

The application of HOST is a valuable tool for the routine identification and selection of viable spermatozoa for ICSI (Stanger et al., 2010). To date, most patients with PCD are reported to have 100% immotile sperm (Matsumoto et al., 2010). Consistent with that report, no motile sperm were found in the ejaculated specimen in our case. While, the use of unselected immotile sperm from PCD patients is usually associated with fairly low fertilization rates owing to the high chance of selecting non-viable sperm for ICSI (Alosilla Fonttis et al., 2002), HOST is a simple and valuable tool for selecting spermatozoa with a functionally intact plasma membrane with improved fertilization rates (Peeraer et al., 2004). Casper et al. (1996) also showed that the HOST can be used successfully to select viable spermatozoa and increase the fertilization rate nearly twofold over random selection (Casper et al., 1996). In summary, we report a successful fertilization outcome via ICSI with HOST activated sperm from a patient carrying two novel *CCDC40* mutations from a Chinese PCD family, we also found diverse ultrastructural defects in cilia of the different patients carrying the same mutations, which were not reported previously in PCD patients.

## Author Contributions

JW, XS, SB, and LY designed and coordinated the study. JC, XB, ZP, and HC assessed the clinical findings of the cases. HH, PH, SF, and NY performed the molecular genetic studies and analyzed the data. SB and LY wrote the draft of the manuscript with input from the other co-authors. All authors read and approved the final manuscript.

## Conflict of Interest Statement

The authors declare that the research was conducted in the absence of any commercial or financial relationships that could be construed as a potential conflict of interest.

## Footnotes

1 http://www.mutationtaster.org/

2 http://www.umd.be/HSF3/

## Supplementary Material

FIGURE S1 Analysis of the EX17_20 deletion region using IGV.

FIGURE S2 Location of two mutations we detected in *CCDC40*. Eight coiled coil domains in the *CCDC40* protein are indicated by blue rectangles (amino acid numbers shown in rectangles). The mutations we detected occur in four coiled coil domains regions (2,6,7,8), including c.1259delA (p.Val421TrpfsX2) and EX17_20 deletion (in red); nine mutations identified in the previous studies indicated in black. EX17_20 deletion resulted the loss of amino acids from 904 to 1142.

DATA SHEET S1 The list of known genes associated with PCD in OMIM.

DATA SHEET S2 All the detected variations in the 20 known genes.

DATA SHEET S3 Complement instructions of methods.
