# Corrigendum: Compound Heterozygous Variants in the Coiled-Coil Domain Containing 40 Gene in a Chinese Family With Primary Ciliary Dyskinesia Cause Extreme Phenotypic Diversity in Cilia Ultrastructure

**DOI:** 10.3389/fgene.2018.00109

**Published:** 2018-04-04

**Authors:** Lin Yang, Santasree Banerjee, Jie Cao, Xiaohong Bai, Zhijun Peng, Haixia Chen, Hui Huang, Peng Han, Shunyu Feng, Na Yi, Xueru Song, Jing Wu

**Affiliations:** ^1^Centre for Reproductive Medicine, Tianjin Medical University General Hospital, Tianjin, China; ^2^BGI Genomics, BGI-Shenzhen, Shenzhen, China; ^3^Department of Respiratory, Tianjin Medical University General Hospital, Tianjin, China; ^4^Department of Radiology, Tianjin Medical University General Hospital, Tianjin, China

**Keywords:** CCDC40, PCD, targeted next-generation sequencing, mRNA expression, intracytoplasmic sperm injection

In the original article, there were two mistakes in the sixth paragraph of the Discussion. The words “immmotive” and “sperm flagella” have been replaced with “immotile” and “bronchial ciliary” respectively. The corrected paragraph appears below.

In addition, no significant change in the length of cilia was identified in respiratory cilia from PCD patients carrying mutations in *CCDC40* gene, implying that mutant *CCDC40* protein disrupted ciliary movement by gross ultrastructural defects (Becker-Heck et al., 2011). It is evident from the cross-section, that abnormal changes in the morphology of the sperm flagella are the most serious (**Figure 5**), resulting in sperm that is 100% immotile. By contrast, cilia with normal structure emerge in specimens from three different level (the first, second, third bronchial levels) of the siblings. From the longitudinal section, the bronchial ciliary axoneme structure getting worse from the proximal to distal (**Figures 4B–E**), but the microtubules of the bronchial cilia are relatively fixed (**Figures 4A–F**), such structures, though abnormal, may have limited impact on ciliary coordination. Therefore, the abnormal performance of the respiratory system of the proband is much less than that of the reproductive system. Adults with PCD is heterogeneous and usually moderate in respiratory function, but appears more severe in women (Frija-Masson et al., 2017). The abnormality of lung function in the siblings is similar, but the sister is heavier than proband in bronchiectasis and lung infection. Compared to the proband (**Figures 3A–C**), the congestive edema of mucous membrane of the elder sister is more apparent (**Figures 3D–F**) with more purulent exudate and larger amount of purulent secretion are secreted in the tracheal carina (**Figure 3F**). To our surprise, the bronchial ciliary electron microscopy performance of the sister was slightly better than the proband; we speculate that this is because the ciliary membrane protrudes outward and extends into a tubular structure. This phenomenon has not been reported in the literature.

The authors apologize for theses mistakes and state the they do not change the scientific conclusions of the article in any way.

The original article has been updated.

## Conflict of interest statement

The authors declare that the research was conducted in the absence of any commercial or financial relationships that could be construed as a potential conflict of interest.
