# Supplementary figures and images for: Compound Heterozygous Variants in the Coiled-Coil Domain Containing 40 Gene in a Chinese Family with Primary Ciliary Dyskinesia Cause Extreme Phenotypic Diversity in Cilia Ultrastructure
Source: Front Genet. 2018 Feb 2;9:23. doi: 10.3389/fgene.2018.00023 (PMC5801289; doi:10.3389/fgene.2018.00023)

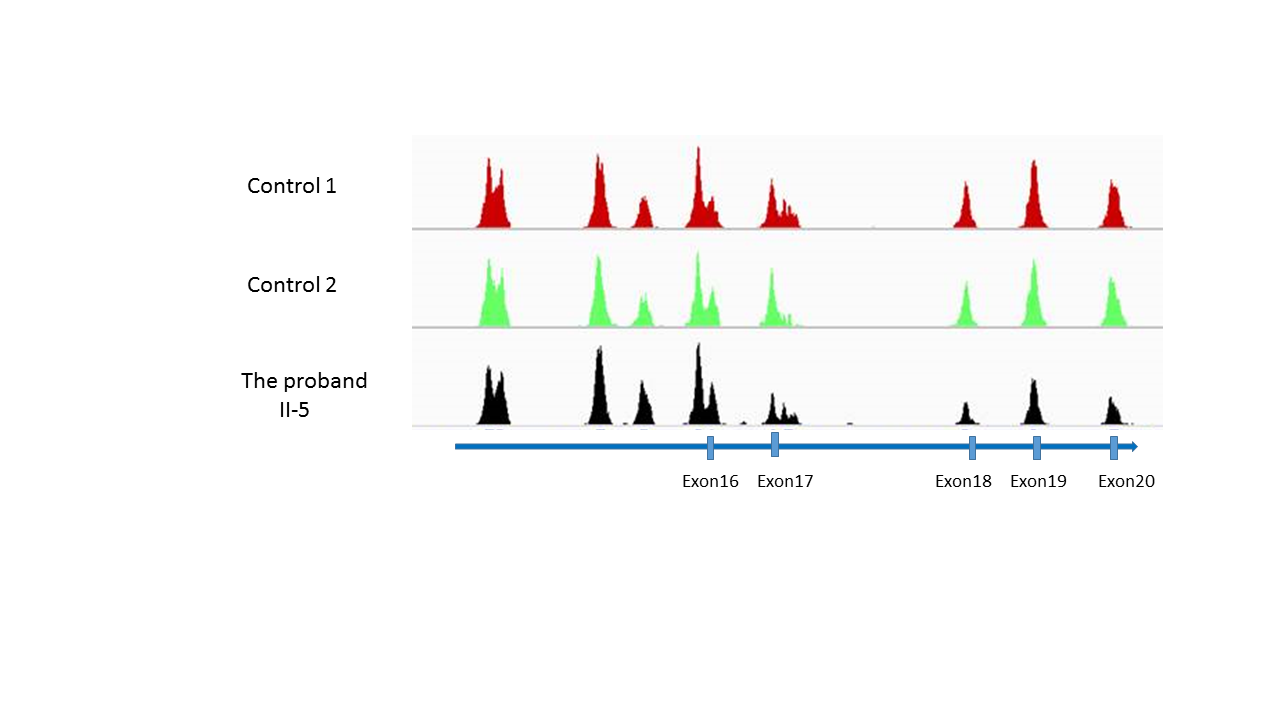

Supplement: FIGURE S1 — Analysis of the EX17_20 deletion region using IGV. [file Image_1.TIF]

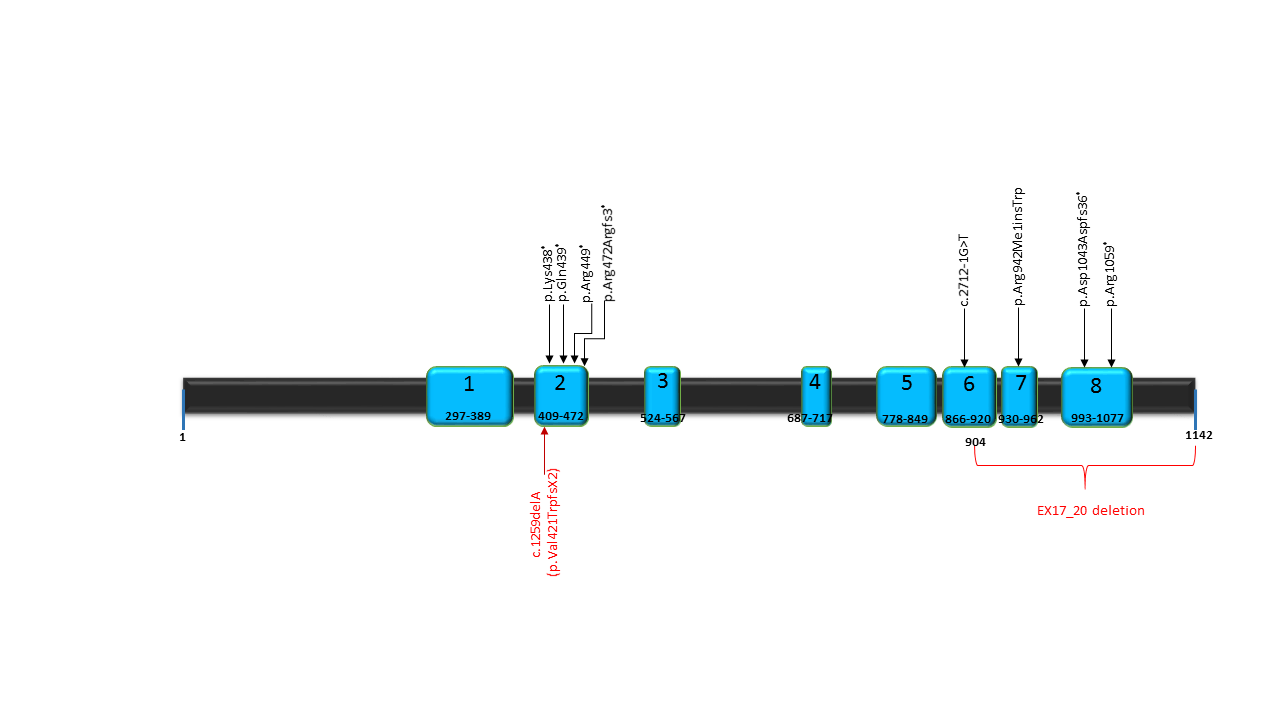

Supplement: Supplementary file 5 [file Image_2.tif]
