# Supplementary material for: Compound Heterozygous Variants in the Coiled-Coil Domain Containing 40 Gene in a Chinese Family with Primary Ciliary Dyskinesia Cause Extreme Phenotypic Diversity in Cilia Ultrastructure
Source: Front Genet. 2018 Feb 2;9:23. doi: 10.3389/fgene.2018.00023 (PMC5801289; doi:10.3389/fgene.2018.00023)
Supplement: DATA SHEET S3 — Complement instructions of methods. [file Data_Sheet_3.DOC]

**Complement Instructions of Methods**

| **Methods Name** | **Complement instructions** |
| --- | --- |
| **Semen Analysis** | Semen samples were obtained via masturbation after 7 days of sexual abstinence in accordance with the *Laboratory Manual for the Examination and Processing of Human Semen, 5th edition^1^* |
| **Sex hormone Analysis** | About 3ml of fasting peripheral venous blood was extracted from the proband. The serum was extracted after waiting for specimen agglutination, 3000 g centrifugal for 15 min. Serum hormone level measurement was preformed by electrochemical luminescence method, equipment and reagents were purchased from the American Sigma company. All the tests were performed strictly according to the kit instructions. |
| **Fractional exhaled nitric oxide text (FeNO)** | FeNO test was determined using NIOX MINO (Aerocrine AB, Solna, Sweden) according to the guidelines established by the American Thoracic Society. Patients were asked to inhale the maximum amount of air and were then instructed to exhale the air into the valve connected to the analyzer. The flow rate (50 mL/s) was kept constant, and data was recorded after 90s. All these procedures were followed up by the clinician. |
| **Pulmonary ventilation function test** | The pulmonary ventilation function test used the Master Screen PTF (JEAGER, Germany), and the method also following the American Thoracic Society criterions. The patients should breathe quite normally at first, and carried out for an extended period of time. Then inhale rapidly and deeply, when after a maximal inspiration, the patient should exhale forcefully for at least 6 seconds, and the tests must keep 3 times repeat. |

**References:**

1. World Health Organization. Chapter 2: Standard procedures. In WHO Laboratory Manual for the Examination and Processing of Human Semen. 5th ed. Geneva:World Health Organization, 2010.45-86.
